# Supplementary material for: Reliability and validity of the UK Biobank cognitive tests
Source: PLoS One. 2020 Apr 20;15(4):e0231627. doi: 10.1371/journal.pone.0231627 (PMC7170235; doi:10.1371/journal.pone.0231627)
Supplement: S1 File — (PDF) [file pone.0231627.s001.pdf]

S1 File. Supplementary materials for

Reliability and validity of the UK Biobank cognitive tests.

Supplementary Table 1. Cognitive tests administered at each UK Biobank study.

UK Biobank test	Cognitive domain	Baseline<br>n = 502,536	Repeat<br>n = 20,346	Web-based<br>n = 104,055-111,074	Imaging<br>n = 37,102[a]
Prospective Memory	Prospective memory	Yes: sub-sample<br>n = 225,926	Yes	No	Yes
Pairs Matching	Visual declarative memory	Yes	Yes	Yes<br>n = 110,561	Yes
Numeric Memory	Working memory	Yes: sub-sample<br>n = 51,808	No	Yes<br>n = 111,074	Yes: sub-sample<br>n = 22,904[a]
Fluid Intelligence	Verbal and numerical reasoning	Yes: sub-sample<br>n = 168,415	Yes	Yes<br>n = 108,163	Yes
Reaction Time	Processing speed	Yes	Yes	No	Yes
Trail Making Test	Executive function	No	No	Yes<br>n = 104,055	Yes: sub-sample<br>n = 22,032[a]
Symbol Digit Substitution	Processing speed	No	No	Yes<br>n = 115,237	Yes: sub-sample<br>n = 21,846[a]
Picture Vocabulary	Vocabulary	No	No	No	Yes: sub-sample<br>n = 21,782[a]
Paired Associate Learning	Verbal declarative memory	No	No	No	Yes: sub-sample<br>n = 22,032[a]
Matrix Pattern Completion	Non-verbal reasoning	No	No	No	Yes: sub-sample<br>n = 21,775[a]
Tower Rearranging	Executive function	No	No	No	Yes: sub-sample<br>n = 21,579[a]

Source: http://biobank.ndph.ox.ac.uk/showcase/

[a]As of 12th April 2019. Data collection ongoing.

Supplementary Methods

UK Biobank cognitive test battery

UK Biobank Pairs Matching Test (UKB Pairs Matching). The UKB Pairs Matching test was used to assess visual memory. Before starting the test, participants were shown a demonstration video. In this test, participants were shown a screen with pairs of matching cards arranged randomly in a grid on the screen. The pairs of cards were then turned face down and the participant was to touch the cards to match up all the pairs of cards in the fewest number of touches. When the participant correctly identified two matching cards, the cards disappeared from the screen. If the participant made a mistake, the cards were turned face down again and they continued. All participants completed two trials. In the first trial, 6 cards consisting of 3 pairs were shown. In the second trial, 12 cards consisting of 6 pairs where shown. The score for each trial was the number of errors made before matching all pairs. If participants made 2 or fewer errors on the second trial (12 cards; 6 pairs), they completed a third trial with 16 cards consisting of 8 pairs. For the current study we used only the number of errors made on the second trial (12 cards; 6 pairs). This was because the first trial (6 cards; 3 pairs) was very easy and few participants made errors, and because the third trial (16 cards; 8 pairs) was only completed by a subsample of participants.

UK Biobank Reaction Time Test (UKB RT). UK Biobank created a Go/No-Go test to measure reaction time. Before the test began, participants were shown a video demonstration. For each trial, participants were shown two cards with a symbol on them, side-by-side on the screen. The two cards either had matching symbols, or different symbols on them. Participants were instructed that, when the cards matched, they were to push a button-box that was on the desk in front of them as quickly as possible. When the cards were different, the participant was to do nothing, and, after a short delay, the cards disappeared and a new pair would appear. There were 12 trials in total. The first 5 trials (rounds 0-4) were regarded as practice trials. Of the remaining 7 trials, 4 contained

matching cards. The score is the mean time, in milliseconds, to press the button-box for these four trials with matching cards. For this test, UK Biobank provided us with the same button-box as used at the UK Biobank clinic.

UK Biobank Prospective Memory Test (UKB Prospective Memory). Prospective memory was assessed using a one-trial task. At the start of the UK Biobank cognitive test battery, participants were shown a screen with the following instructions: “At the end of the games we will show you four coloured symbols and ask you to touch the Blue Square. However, to test your memory, we want you to actually touch the Orange Circle instead.” Participants then completed all other tests in the UK Biobank cognitive test battery. At the end of the UK Biobank tests, participants were shown a screen with four shapes (blue square, pink star, grey cross, orange circle) and were instructed to touch the Blue Square. If participants touched the Orange Circle (correct answer) the test ended. If the participant touched any other shape, they received the following prompt: “At the start of the games we asked you to remember to touch a different symbol when this screen appeared. Please try to remember which symbol it was and touch it now.” If the participant touched the blue square, the prompt was repeated (ad infinitum). If the participant touched any other shape, the test ended. In this study, the score we used was a dichotomous variable, coded 1 if the participant correctly touched the orange circle on the first attempt, and 0 if they touched any other shape.

UK Biobank Fluid Intelligence Test (UKB Fluid IQ). The UKB Fluid IQ test was designed to assess verbal and numerical reasoning. This task involved participants answering 13 multiple-choice questions. For each question, the question was presented at the top of the screen and between three and five multiple-choice options were presented underneath. The participant was to select which of the possible options they thought was the correct answer, or select “Do not know” or “Prefer not to answer”. The questions assessed verbal (e.g., “Bud is to flower as child is to…?” Possible answers: Grow/Develop/Improve/Adult/Old) and numerical (e.g.,

“150…137…125…114…104… What comes next?” Possible answers: 96/95/94/93/92) reasoning abilities. The score was the number of questions answered correctly in two minutes.

UK Biobank Numeric Memory Test (UKB Numeric Memory). A backward digit span task was used to assess working memory. Participants watched an instructional video demonstration before they started the task. Participants were briefly shown a two-digit number on the screen. The number then disappeared and, after a short delay, the participants were asked to enter the number in the reverse order using a keypad on the screen. The to-be-remembered digit sequence then became one digit longer each time the participant correctly remembered the digits in the reverse order. If the participant incorrectly remembered a sequence, they were then asked to recall a different number sequence of the same length. The test ended when the participant incorrectly recalled two trials of the same digit length, or once the participant correctly remembered a 12-digit number. The score was the maximum number of digits correctly remembered in the reverse order. We note that, for this test, all of the to-be-remembered digit-sequence appeared on the screen at once (e.g., participants saw “48951” on the screen). Therefore it was possible to get the correct answer either by carrying out a backward digit span (e.g., reading the numbers left-to-right and mentally reversing the digits) or a forward digit span (e.g., reading from right-to-left and remembering “15981”).

UK Biobank Trail Making Test (UKB TMT). The UKB TMT is a computerised version of the popular Halstead-Reitan Trail Making Test [1], often described as a test of executive function. This test consisted of two parts and each part started with a practice test. In part A, participants were shown a screen with the numbers 1-25 arranged pseudo-randomly on it. The participants were instructed to touch the numbers in numerical order. In part B, the numbers 1-13 and the letters A-L were arranged pseudo-randomly on the screen. Participants were instructed to switch between touching the numbers in numeric order and letters in alphabetical order (i.e., 1-A-2-B-3-C). For each part, participants were instructed to work as quickly and as accurately as possible. If the participant touched an incorrect number or letter, the screen flashed red to inform the participant that they had

made a mistake and the participant was required to touch the correct number or letter before they could move on. The score was the time, in deciseconds, to complete each part.

UK Biobank Symbol Digit Substitution Test (UKB Symbol Digit). A substitution test was used to measure processing speed. This test is similar to other commonly-used substitution tests including the Wechsler Adult Intelligence Scale IV (WAIS-IV) Coding test [2] and the Symbol Digit Modalities Test [3]. Participants were shown a key which paired symbols (top row) with numbers (bottom row). Underneath the key was a row of symbols. The participant's task was to enter the number, using a keypad on the screen that is paired with the symbol. Before starting the test proper, participants completed a practice trial. Participants were instructed to work as quickly and accurately as possible. The score was the number of correct symbol-digit matches made in 60 seconds.

UK Biobank Picture Vocabulary (UKB Picture Vocabulary). To measure vocabulary, an adapted version of the NIH Toolbox Picture Vocabulary test [4] was used. In the UKB Picture Vocabulary test, participants were shown a screen with four pictures and a written word underneath. The participant was instructed to click on which of the pictures was most closely related to the written word. The items varied in difficulty. To reduce the administration time, this test used computerised adaptive testing which means that the difficulty of the items presented to the participant depended on the correctness of their answers to previous items. Before starting the test proper, participants completed two practice items. The score was an estimate (theta score) of the participant's level of vocabulary, and it could range from -11 to 11, with higher scores representing better vocabulary.

UK Biobank Paired Associate Learning (UKB PAL). An adapted version of the Paired Associate Learning test from Test the Nation [5] was used to measure verbal declarative memory. Participants were instructed that they were going to see a screen containing word pairs and that they were to try to remember the word pairs as they will be tested on them later. Participants were then presented with a screen containing twelve pairs of words for 30 seconds (learning phase). After the learning phase, the participants completed another test (the UK Biobank Matrix Pattern test). Then,

participants were presented with the first word of a pair (the target word) and were asked to select, from a list of four options, which word was paired with the target word, or select “Prefer not to answer”. There were 10 questions in total. The score was the number of questions answered correctly.

UK Biobank Tower Rearranging Test (UKB Tower Test). An adapted version of the One-touch Tower of London test [6] was used to assess planning abilities, often described as a component of executive function. In this test, participants were shown a display (display A) with three pegs on it and three different coloured hoops placed on the pegs. Underneath display A was another display (display B). Display B again had three pegs and the same three coloured hoops; however, the hoops were arranged in different locations to that shown in display A. The participant’s task was to work out the number of moves that it would take to make display A look like display B. The number of moves required for each item could range between 1 and 6. The score was the number of items answered correctly in 3 minutes.

UK Biobank Matrix Pattern Completion (UKB Matrices). An adapted version of the COGNITO Matrices test [7] was used as a measure of non-verbal fluid reasoning. For each item, participants were presented with a matrix design, with a piece missing in the lower right hand corner. The pattern has a logical structure, which can be used to work out what the missing piece should look like. The task was to work out the rules of the patterns and select, from a list of between 6 and 8 alternatives, the correct missing piece of the puzzle. Participants completed two practice examples before starting the test proper. The items ranged in difficulty from easy to difficult. There were 15 items in total, and the score was the number of items answered correctly in 3 minutes.

General tests

Cambridge Examination of Mental Disorders of the Elderly (CAMDEX) Self-rated memory [8]. To assess subjective memory complaints, the tester asked participants four questions about their self-reported memory. The questions assessed general memory problems, difficulties remembering

where they left common objects, difficulties remembering names, and whether participants have ever become lost in a familiar environment. For each question participants answered yes (scored 1) or no (scored 0). A higher score reflected poorer self-rated memory.

Mini Addenbrooke's Cognitive Examination UK Version B [9] (M-ACE). The M-ACE is a 30-item abbreviated version of the Addenbrooke's Cognitive Examination III [10]. The test assesses attention (orientation in time), memory (immediate and delayed memory of a name and address), fluency (animal naming), and visuospatial abilities (clock drawing). This test has been validated in dementia patients and has been found to be more sensitive and less prone to ceiling effects than the commonly used Mini-Mental State Examination [11].

Reference tests

Peabody Picture Vocabulary Test, Fourth Edition [12] (PPVT). The PPVT was chosen as a reference test for UKB Picture Vocabulary. Participants were shown a page with four pictures on it and the tester read out a word. The participant's task was to decide which of the four pictures shows the meaning of the word. Prior to beginning the test proper, participants were presented with two practice items. The test consisted of 19 sets of 12 words (228 items). Each set of words was progressively harder than the last. Participants started at set 14 (item number 157). If the participant made one or fewer errors on set 14, participants moved on to set 15 and full marks were awarded for sets 1-13. If two or more errors were made in set 14, lower sets were administered (in reverse order), until the participant made one or fewer errors in a set. Again, full marks were awarded for easier sets not administered. The test ended when participants completed set 19, or when participants made 8 or more errors on a set. One point is given for each correctly answered question. The scores is the number of questions answered correctly.

National Adult Reading Test [13] (NART). The NART was selected as a reference test for UKB Picture Vocabulary. Participants were given a piece of paper with 50 irregularly-pronounced words written on it. The participant was asked to read the words aloud. One cannot rely on the spelling to correctly

pronounce the word; therefore, prior knowledge of the word is required to know how to pronounce it correctly [13]. One point was given for each correctly pronounced word. This brief test is often used as a measure of premorbid ability because it has been found to be robust to the effects of cognitive decline [14, 15].

NIH Toolbox Picture Vocabulary [4]. To compare the UKB Picture Vocabulary to the original version of this test, participants were administered the NIH Toolbox Picture Vocabulary. The UKB Picture Vocabulary uses the same visual stimulus and words as in the original NIH Toolbox Picture Vocabulary test. This test was administered on an iPad tablet computer. The participant was presented with a screen with four pictures on it and heard a spoken word. The word was spoken with an American accent in the NIH Toolbox Picture Vocabulary test. Like the UK Biobank version, this original version was adaptive and therefore the items presented depended on the correctness of participant's previous answers. The score was an estimate (theta score) of the participants level of vocabulary. Higher scores reflected a higher vocabulary level.

Rivermead Behavioural Memory Test - Extended Version, Appointments [16] (RMBM Appointments). The RMBM Appointments test was selected as a reference test for UKB Prospective Memory. In this test, participants were informed that the tester was going to set the alarm to ring in 20 minutes time. Before starting the test proper, the tester played the alarm to let the participant know what the alarm sounded like. The tester then set the alarm for 20 minutes. The participant was instructed that when the alarm rings, they should ask the tester 2 questions ("When do I have to see you again?" and "When does this session end?"). When the alarm sounded, participants who spontaneously asked the two questions were awarded 2 points per question (max score = 4). If the participant did not spontaneously ask the tester the two questions, the tester prompted the participant by asking them "What [else] were you going to do when the alarm rings?" If the participant provided the questions following the prompt, 1 point was awarded per question.

Wechsler Memory Scale IV (WMS-IV) Designs [17]. The WMS-IV Designs test was chosen as a reference test for the UKB Pairs Matching test. This is a test of visuospatial memory and it consists of two parts: Designs I and Designs II. In Designs I, a blank 4x4 grid was placed in front of the participant. Participants were then presented with a page containing a 4x4 grid with between 4 and 8 abstract designs in some of the cells of this grid. The page was shown for 10 seconds and then removed. A pack of cards with designs on them was then handed to the participant. The participant's task was to select from the pack of cards which designs were on the page, and then place the designs on the grid in the same configuration as on the previous page. A practice example using two designs was used to help participants understand the task. There were 4 trials in total; one containing 4 designs, two containing 6 designs, and one containing 8 designs. Points were awarded if participants selected the correct design (2 points awarded for selecting the correct design, 1 point awarded for selecting the distractor design), placed a design in the correct location (1 point awarded if participants placed *any* design in one of the correct locations), and placed the correct design in the correct location (2 points awarded if correct design was placed in the correct location). The maximum score for Designs I was 120 points.

Approximately 20 minutes after completing Designs I, in which participants completed other cognitive tests, Designs II was administered. The 4x4 grid was placed in front of the participant. For each trial, participants were presented with a pack of cards and asked to select from the pack which designs were shown earlier and they were asked to place these designs on the grid in the same configuration as on the page shown earlier. The same scoring system as used in Designs I was used for Designs II. The maximum score for Designs II was 120.

WMS-IV Verbal Paired Associates [17] (VPA). WMS-IV VPA is a test of verbal declarative learning and memory and was chosen as a reference test for the UKB Paired Associates Learning test. VPA is made up of two parts: VPA I and VPA II. In the learning phase of VPA I the tester read aloud a list of 14 word-pairs to the participant. Immediately after reading the word-pairs, the tester said the first

word of each pair, and the participant was to recall the word that went with it (test phase). The test consisted of four trials and, for each trial, the same 14 word-pairs were used in the learning and test phase. The order of the word-pairs differed each trial. The score for VPA I is the number of correctly recalled word-pairs across the four trials (maximum score = 56).

Approximately 20 minutes after completing VPA I, VPA II was administered. Participants completed other cognitive tests in the delay between VPA I and VPA II. Without hearing the word-pairs again, the tester read out the first word of each pair and the participant was to recall which word was paired with it. The score was the number of correctly recalled words (maximum score = 14).

WAIS-IV Digit Span [2]. The WAIS-IV Digit Span test is a measure of working memory and it was chosen as a reference test for UKB Numeric Memory. This test is made up of three different tasks. For Digit Span Forwards, the tester read aloud a sequence of numbers to the participant, and the participant was required to immediately recall the numbers in the same order. For Digit Span Backwards, the tester read aloud a sequence of numbers and the participant was required to recall the numbers in the reverse order. For the Digit Span Sequence, the tester read a sequence of numbers and the participant had to recall the numbers in ascending order. For each task, the participants were first read a two-digit sequence. After completing two trials of the same length, the number of digits in the to-be-recalled sequence increased by one. The task ended when the participant incorrectly recalled 2 trials of the same length, or when the participant had completed 16 trials. For Digit Span Backwards and Digit Span Sequence (but not Digit Span Forwards) participants completed two practice trials before starting the test proper. For each task, one point was awarded for each correctly recalled trial (maximum score = 16).

Symbol Digit Modalities Test [3] (SDMT). The SDMT is a measure of processing speed and was chosen as a reference test for the UKB Symbol Digit test. Participants were provided with a piece of paper with a key pairing symbols and numbers at the top of the page. Below the key were rows of boxes with symbols in them and empty boxes underneath. The participant’s task was to write the

number in the empty box that was matched with the symbol. Before starting the test proper, participants completed a practice consisting of 8 symbol-digit matches. The score was the number of correct symbol-digit matches made in 90 seconds.

Deary-Liewald Reaction Time Test [18] (DLRT). The DLRT is a computerised task made up of two parts—Simple reaction time (RT) and Choice RT—and was chosen as a reference test for UKB RT. Both parts began with a practice example consisting of 8 trials. In the Simple RT task, participants were shown a screen with a blue background and a white square in the middle. The task was to push the spacebar on a keyboard as quickly as possible each time a cross appeared in the box. There were 20 trials. The score was the mean time, in milliseconds, to press the spacebar when the cross appeared. In the Choice RT test, there were four white boxes on the computer screen. Participants were instructed to place their index and middle fingers on the "Z", "X", comma and full stop keys on the keyboard. These four keys corresponded to the four boxes on the screen. A cross could appear in any of the four boxes. The participant's task was to press the correct key when a cross appeared in one of the four boxes on the screen as quickly as possible. There were 40 trials. The score was the mean time, in milliseconds, to press the correct key.

Trail Making Test (TMT). The Halstead-Reitan [1] TMT test was used to measure switching ability, often described as a component of executive function. This test was used as a reference test for UKB TMT. This paper-and-pencil test consisted of two parts and each part started with a short practice example that the tester completed to show the participant what they were to do. In part A, participants were provided with a piece of paper with the numbers 1 to 25 arranged on the page. The participant's task was to draw a line trail through each number in numeric order. In part B, participants were provided with a piece of paper with the numbers 1 to 13 and the letters A to L arranged on the page. The participant's task was to switch between joining up the numbers in ascending order and letters in alphabetical order (e.g., 1-A-2-B-3-C). If the participant made a

mistake, the tester would instruct the participant to start from the last correct number or letter. The score used in this study was the time, in seconds, to complete part A and part B.

Delis-Kaplan Executive Function System (D-KEFS) Tower Test [19]. The D-KEFS Tower Test is a test of planning ability—often described as a component of executive function— and was used here as a reference test for the UKB Tower Test. In this test, the participant was given a wooden base with three pegs on it. The tester placed between 2 and 5 wooden disks of varying size on to the wooden pegs. The participant was shown a page with an illustration of the peg board with the discs arranged in the target sequence. The participant's task was to move the wooden discs on the board to match the target display using the fewest possible moves. Participants were instructed to follow two rules: they must move only one disc at a time, and they cannot place a larger disc on top of a smaller disc. There were 9 trials in total that started easy (2 discs) and became progressively harder (up to 5 discs). Each trial had a time limit. For each trial, a score of 0 was given if participants failed to match the peg board to the target sequence or if they ran out of time. Between 1 and 4 points were allocated based on the number of moves taken to correctly make the peg board look like the target display. Higher scores reflect better performance (maximum score = 30).

COGNITO Matrices [7]. The COGNITO Matrices task is a measure of non-verbal reasoning and was used as a reference test for UKB Matrices. UKB Matrices is an adapted version of the COGNITO Matrices test. UKB Matrices uses the same items as the original version. In this computerised test, participants were presented with a matrix design on the computer screen with a piece missing in the lower right hand corner. The participant was to touch which of the puzzle pieces, from a list of 6 to 8 alternatives, completed the design. There were 15 items in total, and each item had a 30 second time limit. If participants had not provided an answer in this time limit, the next question would appear and a score of 0 would be given for that item. Two points were awarded for each correctly answered question, and a score of 0 was awarded for incorrect items (maximum score = 30).

Supplementary Table 2. Test order for participants with even and odd participant identification numbers.

Even participant ID numbers	Odd participant ID numbers
Information sheet and consent	Information sheet and consent
Demographic and health questionnaire	Demographic and health questionnaire
Self-rated memory	Self-rated memory
UK Biobank cognitive test battery: test order	RMBM Appointments (instructions)
UKB Prospective Memory (instructions)	Mini Addenbrooke's Cognitive Examination
UKB Pairs Matching	Peabody Picture Vocabulary Test, Fourth Edition
UKB Numeric Memory	National Adult Reading Test
UKB Fluid Intelligence	RMBM Appointments (test)
UKB Reaction Time	COGNITO Matrices
UKB Trail Making Test	Deary-Liewald Reaction Time
UKB Symbol Digit Substitution	WMS-IV Designs I
UKB Picture Vocabulary	Symbol Digit Modalities Test
UKB Paired Associate Learning (learning phase)	WMS-IV Verbal Paired Associates I
UKB Matrix Pattern Completion	D-KEFS Tower Test
UKB Paired Associate Learning (test phase)	WMS-IV Designs II
UKB Tower Rearranging	Trail Making Test
UKB Prospective Memory (test)	WMS-IV Verbal Paired Associates II
UK Biobank questionnaire[a]	**BREAK (refreshments provided)**
RMBM Appointments (instructions)	NIH Toolbox Picture Vocabulary
Mini Addenbrooke's Cognitive Examination	WAIS-IV Digit Span
Peabody Picture Vocabulary Test, Fourth Edition	UK Biobank cognitive test battery: test order
National Adult Reading Test	UKB Prospective Memory (instructions)
RMBM Appointments (test)	UKB Pairs Matching
**BREAK (refreshments provided)**	UKB Numeric Memory
COGNITO Matrices	UKB Fluid Intelligence
Deary-Liewald Reaction Time Test	UKB Reaction Time
WMS-IV Designs I	UKB Trail Making Test
Symbol Digit Modalities Test	UKB Symbol Digit Substitution
WMS-IV Verbal Paired Associates I	UKB Picture Vocabulary
D-KEFS Tower Test	UKB Paired Associate Learning (learning phase)
WMS-IV Designs II	UKB Matrix Pattern Completion
Trail Making Test	UKB Paired Associate Learning (test phase)
WMS-IV Verbal Paired Associates II	UKB Tower Rearranging
NIH Toolbox Picture Vocabulary	UKB Prospective Memory (test)
WAIS-IV Digit Span	UK Biobank questionnaire[a]

UKB, UK Biobank; RMBM, Rivermead Behavioural Memory Test – Extended Version; WMS-IV, Wechsler Memory Scale – Fourth Edition; D-KEFS, Delis-Kaplan Executive Function System; WAIS-IV, Wechsler Adult Intelligence Scale IV.

[a]Participants who agreed to return for the repeat study visit did not complete the UK Biobank questionnaire during the main testing session. They completed this questionnaire after administration of the UK Biobank test battery at the repeat study visit.

Supplementary Fig 1. Distributions of the UK Biobank tests and the general and reference tests used in this study.

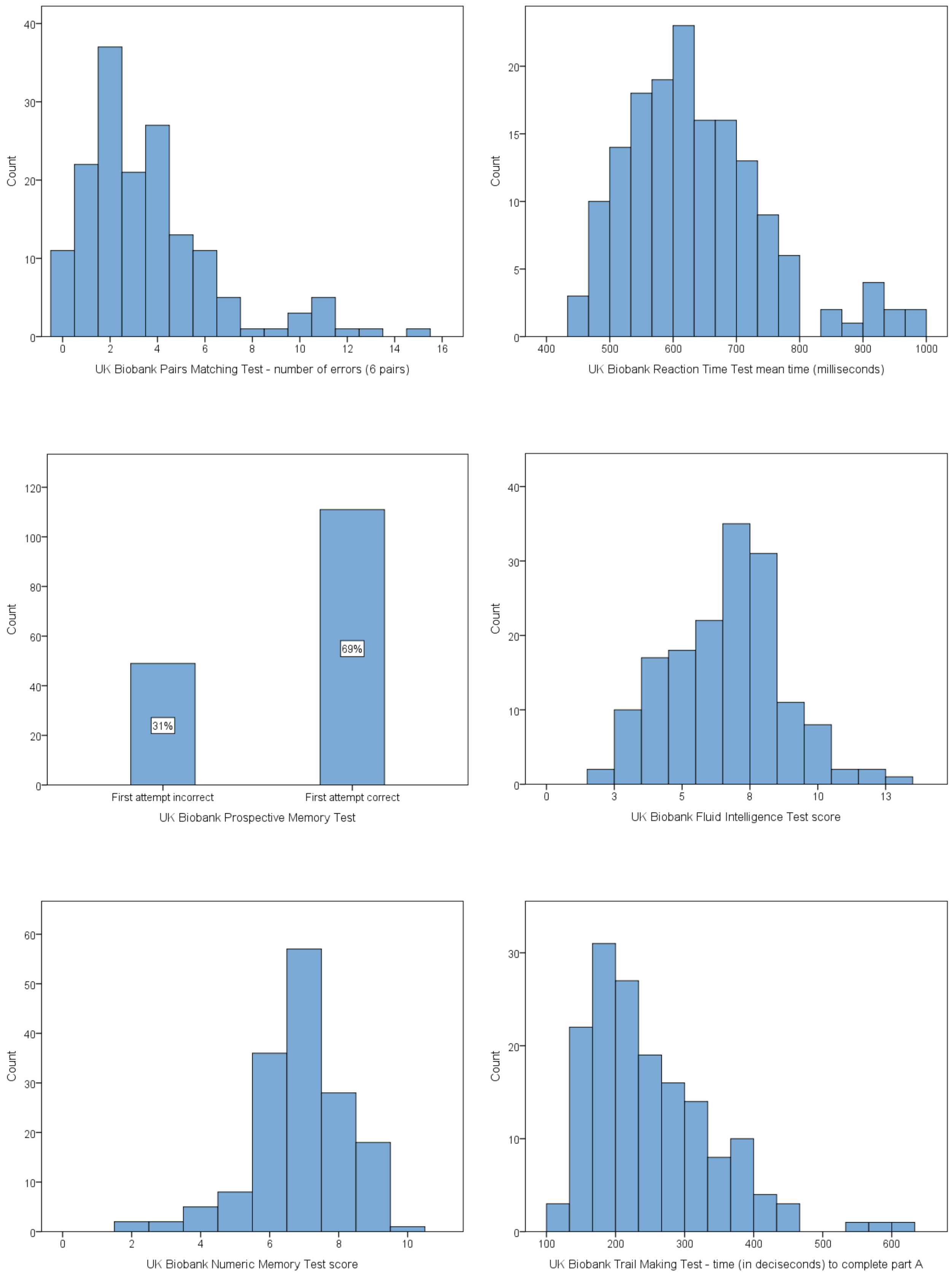

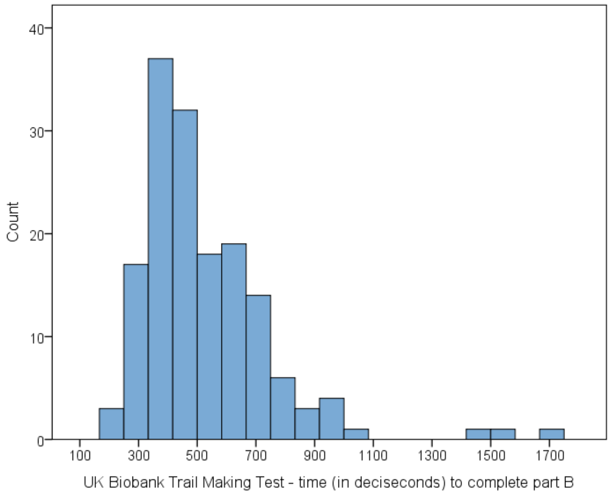
Count
UK Biobank Trail Making Test - time (in deciseconds) to complete part B

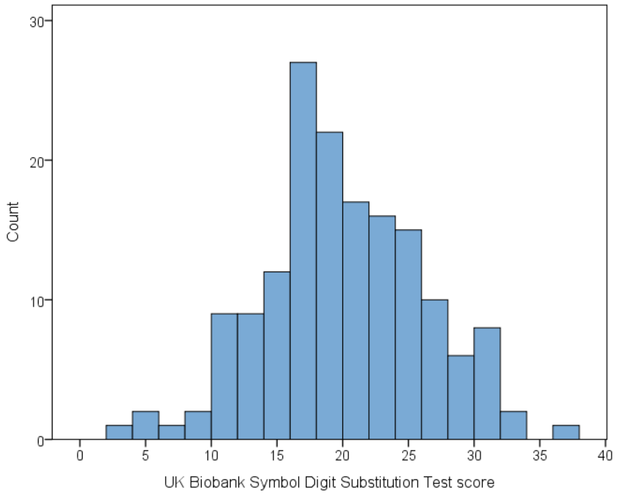
Count
UK Biobank Symbol Digit Substitution Test score

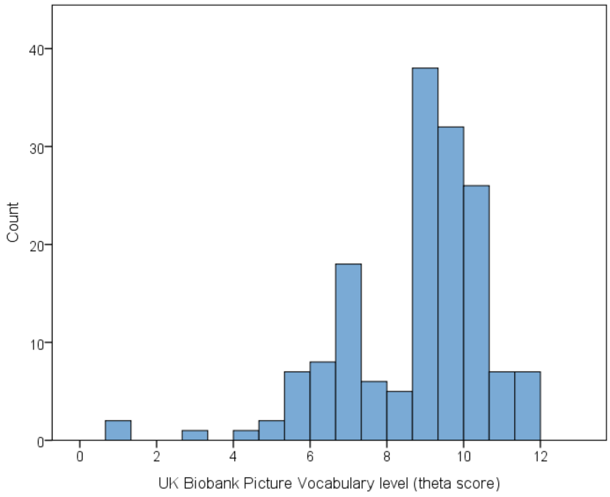
Count
UK Biobank Picture Vocabulary level (theta score)

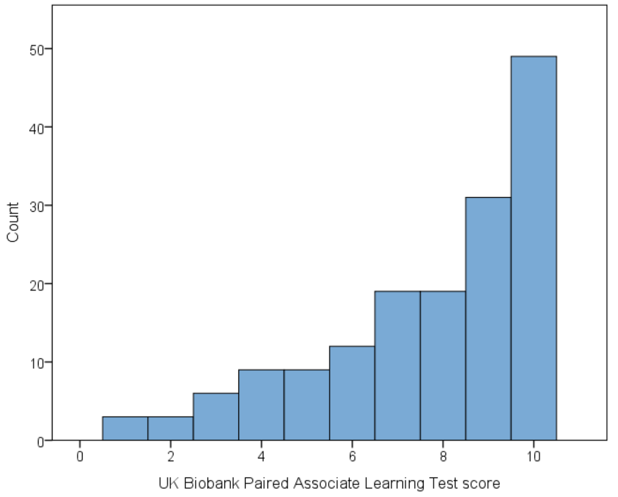
Count
UK Biobank Paired Associate Learning Test score

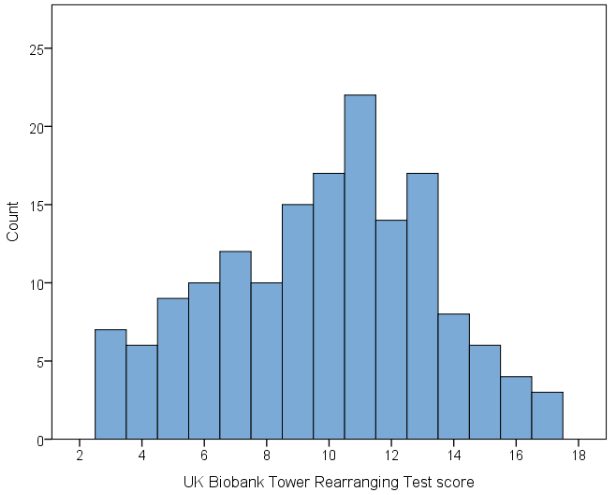
Count
UK Biobank Tower Rearranging Test score

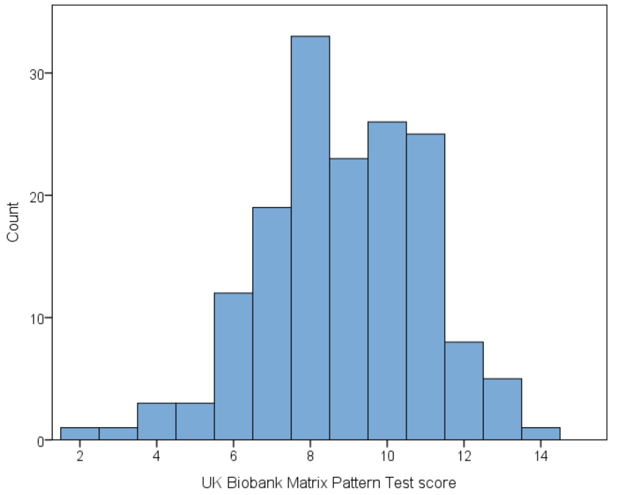
Count
UK Biobank Matrix Pattern Test score

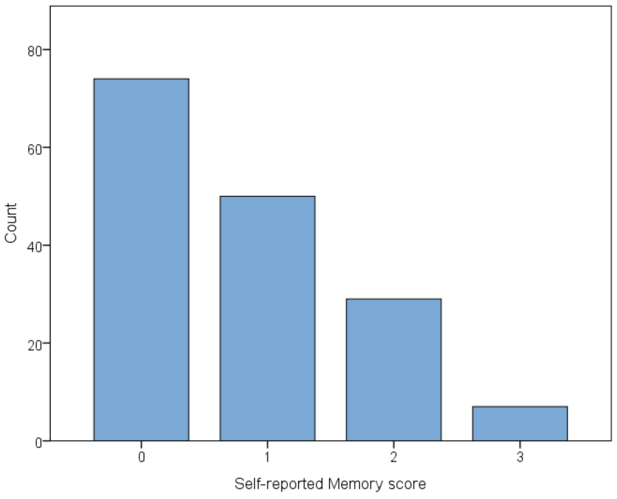
Count
0
20
40
60
80
0
1
2
3
Self-reported Memory score

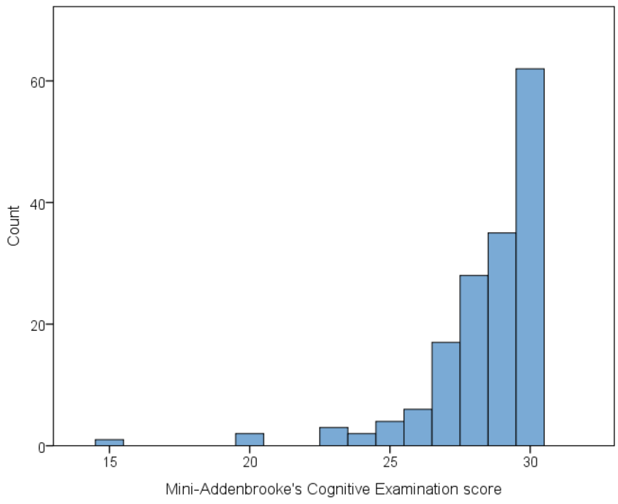
Count
0
20
40
60
15
20
25
30
Mini-Addenbrooke's Cognitive Examination score

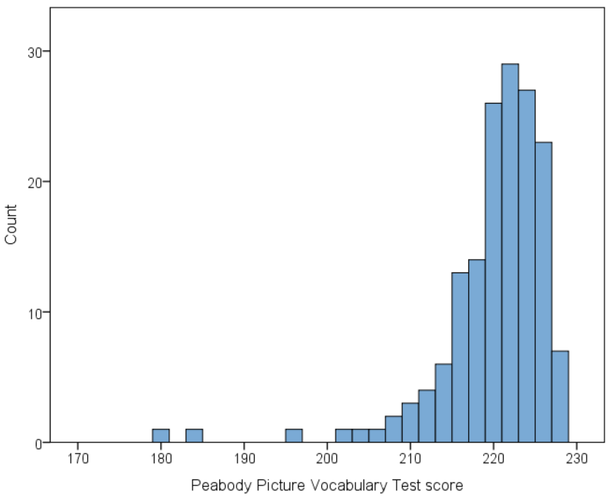
Count
0
10
20
30
170
180
190
200
210
220
230
Peabody Picture Vocabulary Test score

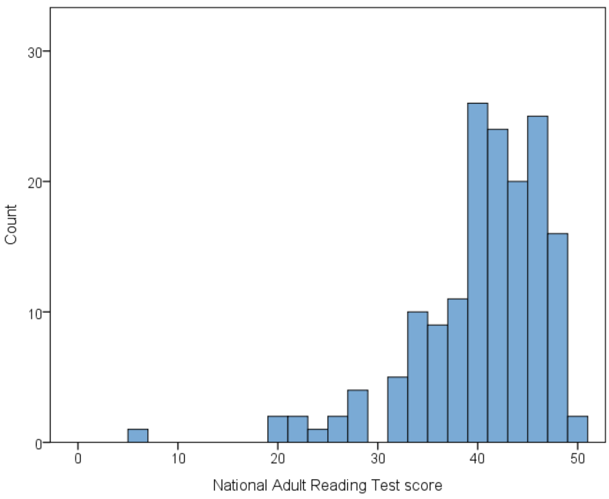
Count
0
10
20
30
0
10
20
30
40
50
National Adult Reading Test score

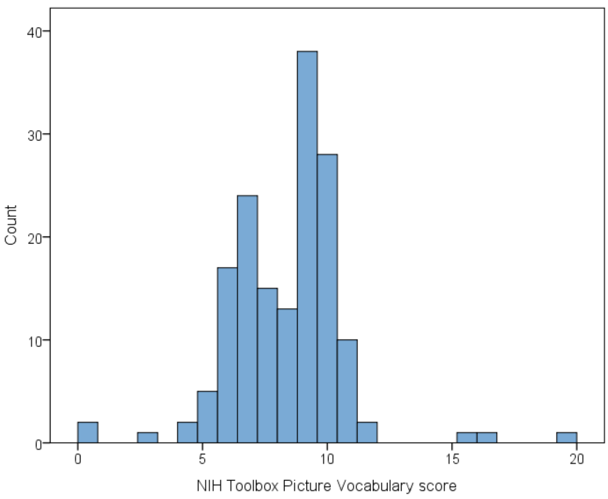
Count
0
10
20
30
40
0
5
10
15
20
NIH Toolbox Picture Vocabulary score

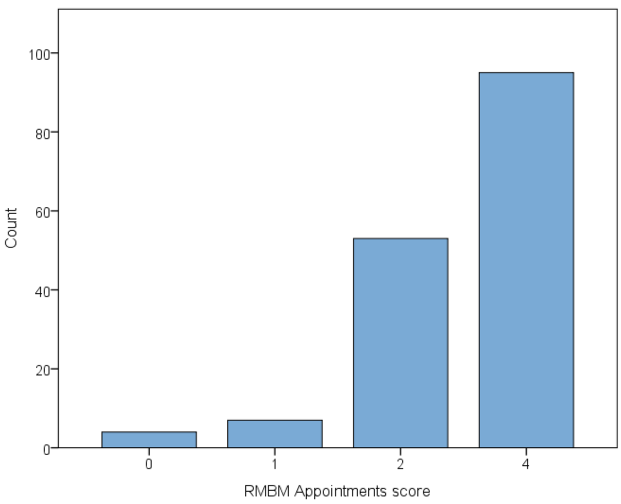
Count
0
20
40
60
80
100
0
1
2
4
RMBM Appointments score

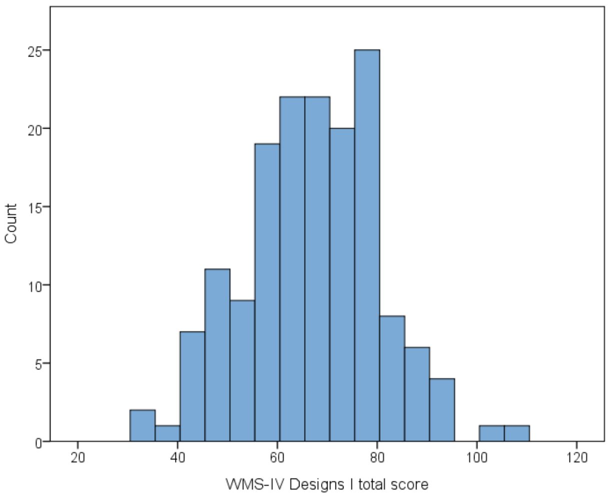
Count
WMS-IV Designs I total score

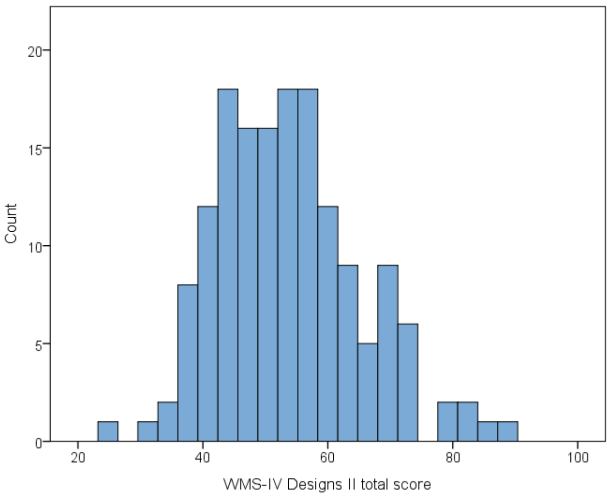
Count
WMS-IV Designs II total score

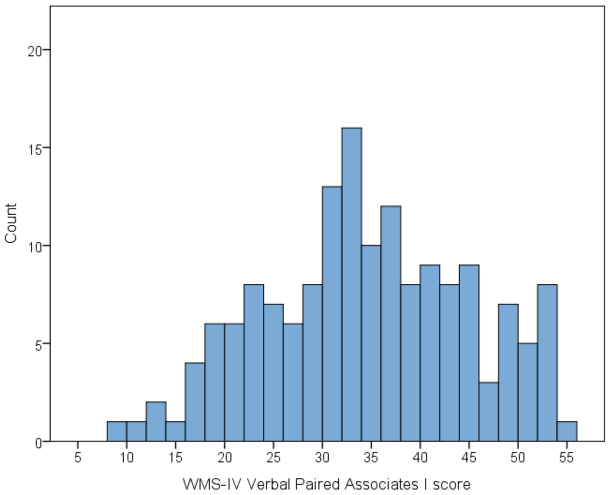
Count
WMS-IV Verbal Paired Associates I score

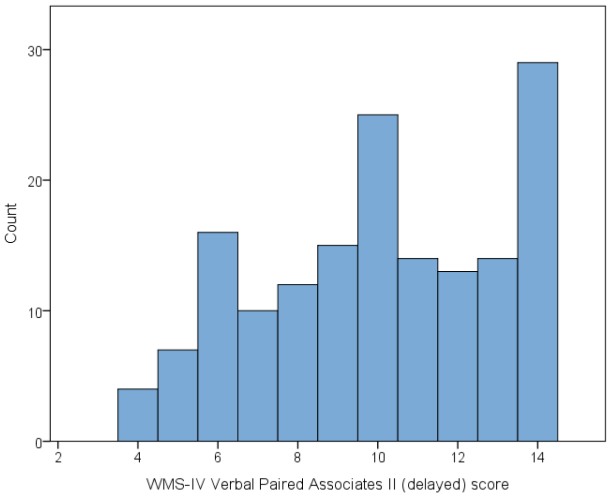
Count
WMS-IV Verbal Paired Associates II (delayed) score

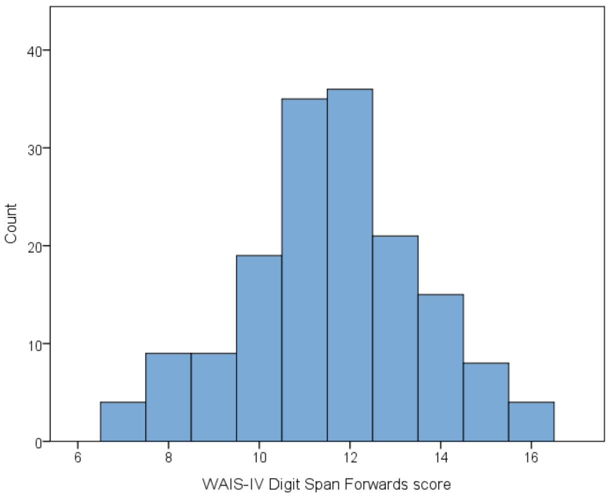
Count
WAIS-IV Digit Span Forwards score

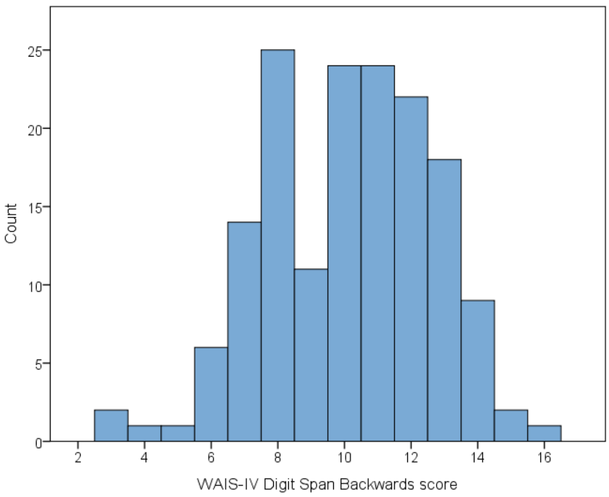
Count
WAIS-IV Digit Span Backwards score

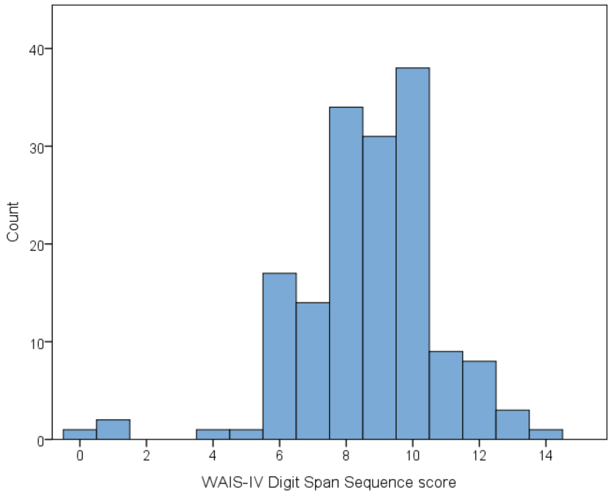
Count
WAIS-IV Digit Span Sequence score

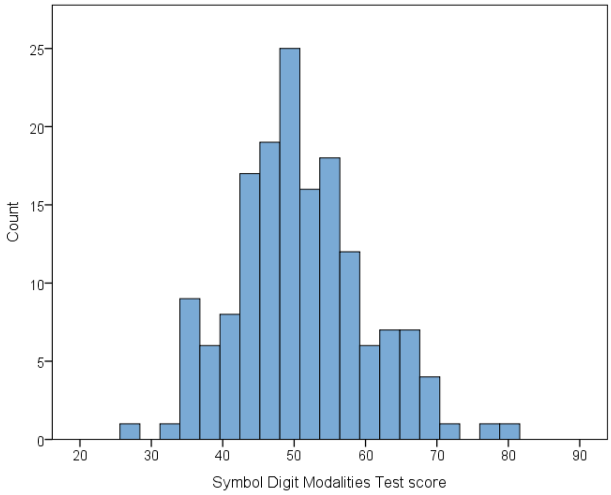
Count
Symbol Digit Modalities Test score

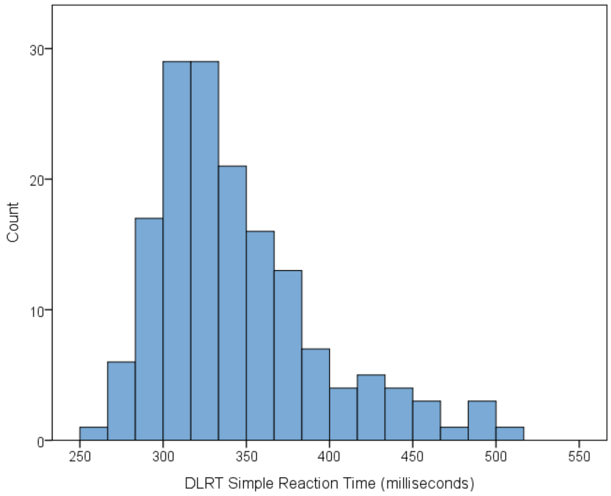
Count
DLRT Simple Reaction Time (milliseconds)

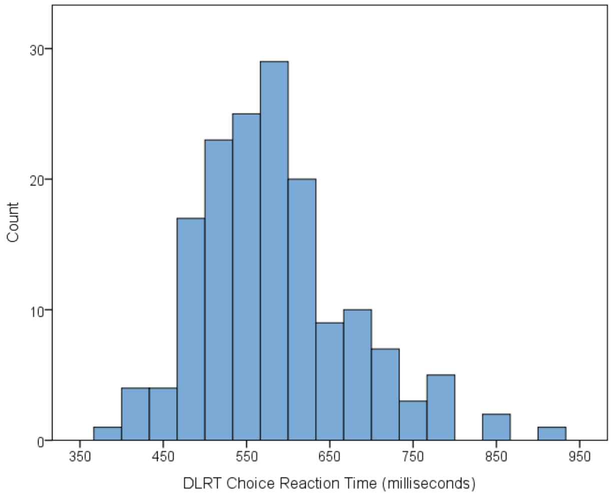
Count
DLRT Choice Reaction Time (milliseconds)

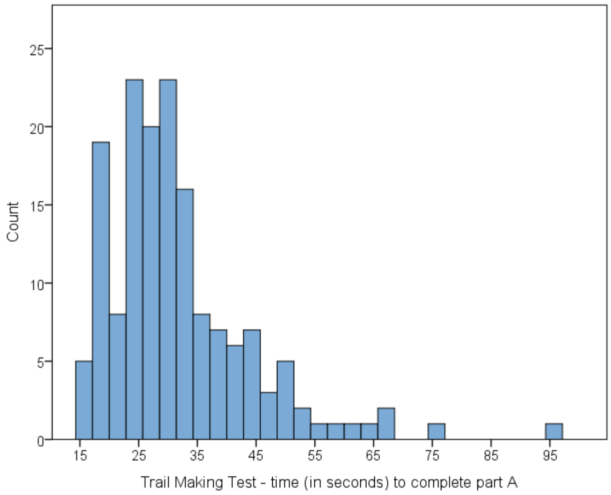
Count
Trail Making Test - time (in seconds) to complete part A

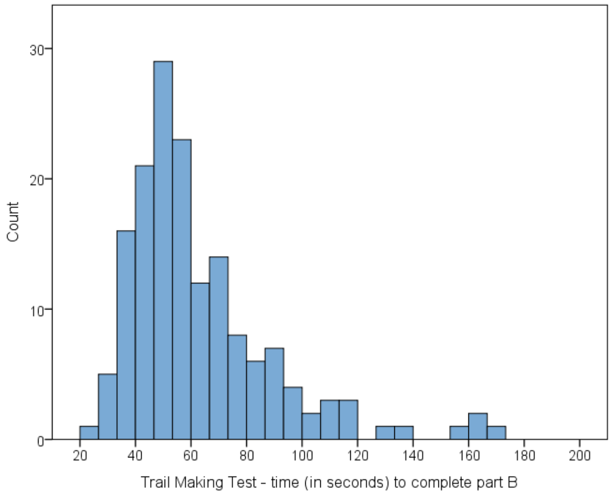
Count
Trail Making Test - time (in seconds) to complete part B

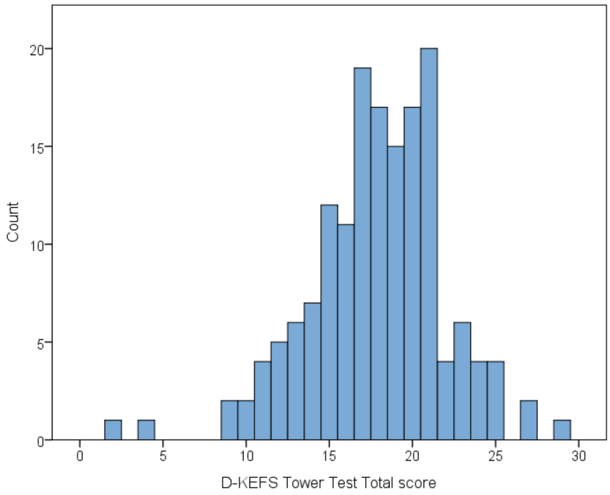
Count
20
15
10
5
0
0
5
10
15
20
25
30
D-KEFS Tower Test Total score

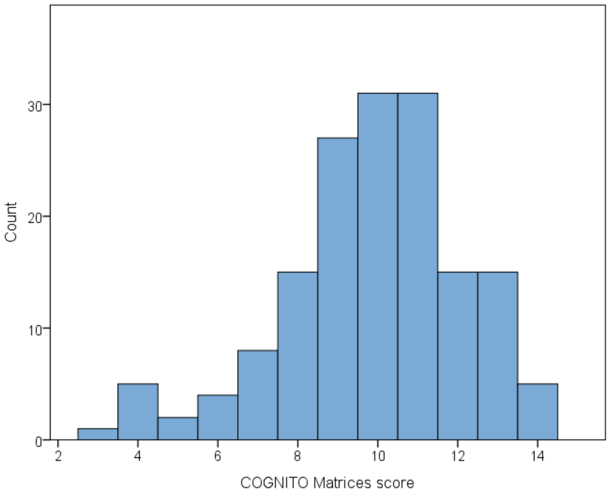
Count
30
20
10
0
2
4
6
8
10
12
14
COGNITO Matrices score

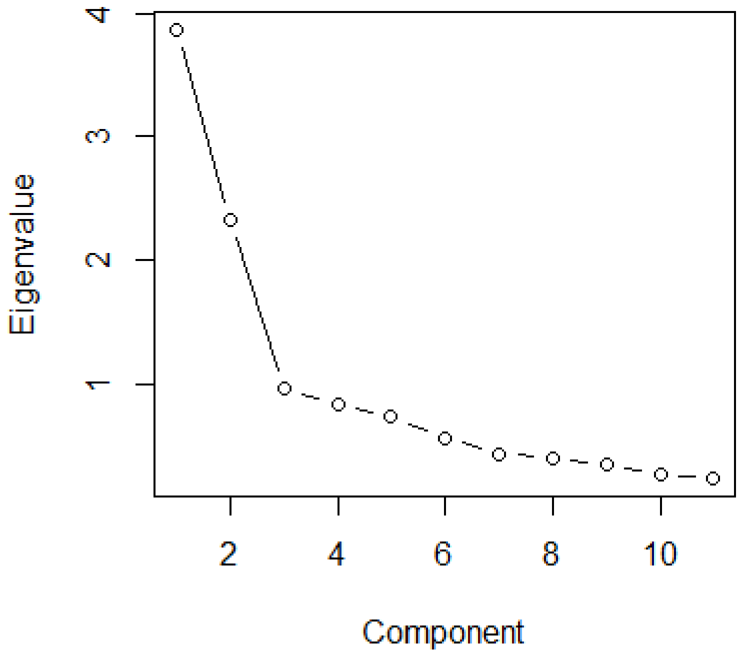

Supplementary Fig 2. Scree plot of eigenvalues from 11 reference cognitive tests.

Supplementary Table 3. Loadings from a principal component analysis of 11 reference cognitive tests (n = 154). Eigenvalues indicated two components. Unrotated and rotated loadings, using oblique (oblimin) rotation, are reported.

	Unrotated PC 1	Unrotated PC 2	Rotated PC 1	Rotated PC 2
Trail Making Test part B	-0.68	0.46	-0.83	0.08
Symbol Digit Modalities Test	0.67	-0.46	0.82	-0.07
WMS-IV Designs Total	0.67	-0.31	0.73	0.06
WAIS-IV Digit Span Total	0.59	0.10	0.42	0.38
D-KEFS Tower Test	0.51	-0.22	0.55	0.06
DLRT Choice Reaction Time	-0.58	0.41	-0.71	0.08
NIH Toolbox Picture Vocabulary	0.51	0.73	-0.02	0.90
National Adult Reading Test	0.44	0.79	-0.12	0.91
PPVT	0.58	0.63	0.10	0.84
WMS-IV Verbal Paired Associates Total	0.59	0.16	0.38	0.43
COGNITO Matrices	0.64	-0.11	0.58	0.22
**Proportion of variance**	**0.35**	**0.21**	**0.31**	**0.25**

PC, principal component; WMS-IV, Wechsler Memory Scale – Fourth Edition; WAIS-IV, Wechsler Adult Intelligence Scale – Fourth Edition; D-KEFS, Delis-Kaplan Executive Function System; DLRT, Deary-Liewald Reaction Time Test; PPVT, Peabody Picture Vocabulary Test – Fourth Edition.

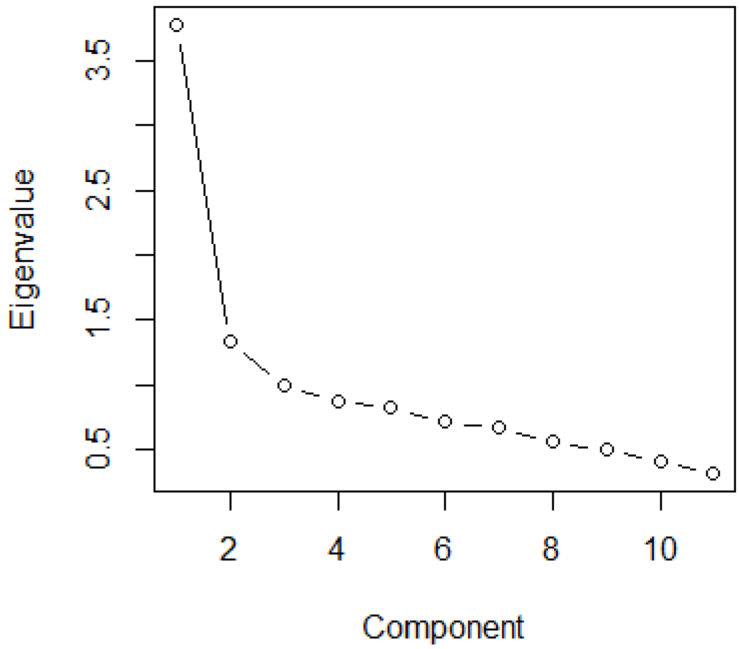

Supplementary Fig 3. Scree plot of eigenvalues from 11 UK Biobank cognitive tests.

Supplementary Table 4. Loadings from a principal component analysis of 11 UK Biobank tests (n = 154). Eigenvalues indicated two components. Unrotated and rotated loadings, using oblique (oblimin) rotation, are reported.

	Unrotated PC 1	Unrotated PC 2	Rotated PC 1	Rotated PC 2
UKB Pairs Matching Test	-0.49	0.22	-0.54	0.11
UKB Reaction Time Test	-0.41	0.08	-0.42	-0.01
UKB Prospective Memory Test	0.54	0.20	0.45	0.32
UKB Fluid Intelligence Test	0.66	0.31	0.52	0.45
UKB Numeric Memory Test	0.58	-0.05	0.57	0.08
UKB Trail Making Test part B	-0.79	0.20	-0.82	0.02
UKB Symbol Digit Test	0.73	-0.31	0.80	-0.15
UKB Picture Vocabulary Test	0.19	0.88	-0.11	0.91
UKB Paired Associate Learning Test	0.49	0.38	0.34	0.48
UKB Tower Test	0.65	-0.28	0.71	-0.13
UKB Matrix Pattern Test	0.66	0.09	0.60	0.23
**Proportion of variance**	**0.34**	**0.12**	**0.33**	**0.14**

PC, principal component; UKB, UK Biobank.

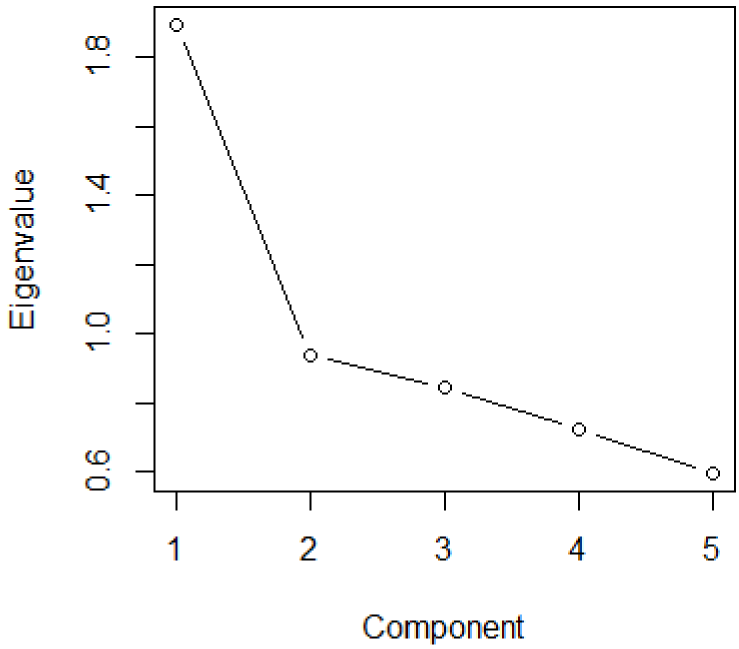

Supplementary Fig 4. Scree plot of eigenvalues from 5 UK Biobank cognitive tests.

Supplementary Table 5. Loadings from a principal component analysis of 5 UK Biobank tests (n = 156). Eigenvalues and scree plot indicated one component. Unrotated loadings are reported.

	Unrotated PC 1
UKB Pairs Matching Test	-0.51
UKB Reaction Time Test	-0.49
UKB Prospective Memory Test	0.64
UKB Fluid Intelligence Test	0.70
UKB Numeric Memory Test	0.70
**Proportion of variance**	**0.38**

PC, principal component; UKB, UK Biobank.

Supplementary Table 6. Participant characteristics for the full sample (n = 160) and for the repeat sample (n = 52).

	Full sample	Repeat sample
Age (years), mean (SD)	62.59 (10.24)	61.69 (9.70)
Sex, n (%)		
Female	94 (58.8%)	24 (46.2%)
Male	66 (41.3%)	28 (53.8%)
Years of full-time education, mean (SD)	16.19 (2.73)	15.52 (2.82)
General health, n (%)		
Poor	1 (0.6%)	0 (0.0%)
Fair	5 (3.1%)	2 (3.8%)
Good	33 (20.6%)	12 (23.1%)
Very good	85 (53.1%)	27 (51.9%)
Excellent	36 (22.5%)	11 (21.2%)
Health compared to one year ago, n (%)		
Much worse now	1 (0.6%)	0 (0.0%)
Somewhat worse now	17 (10.6%)	3 (5.8%)
About the same	115 (71.9%)	42 (80.8%)
Somewhat better now	19 (11.9%)	5 (9.6%)
Much better now	8 (5.0%)	2 (3.8%)

Supplementary Table 7. Descriptive statistics for cognitive tests.

	n	Mean (SD)	Range
UK Biobank tests			
UKB Pairs Matching Test	160	3.59 (2.84)	0-15
UKB Reaction Time Test	159	639.99 (116.70)	436-1029
UKB Prospective Memory Test	160	111 (69.4%)[a]	-
UKB Fluid Intelligence Test	159	6.64 (2.11)	2-13
UKB Numeric Memory Test	157	6.89 (1.39)	2-10
UKB Trail Making Test part A	160	252.29 (91.68)	130-624
UKB Trail Making Test part B	157	527.44 (226.00)	222-1672
UKB Symbol Digit Test	160	19.52 (6.14)	3-36
UKB Picture Vocabulary Test	160	8.81 (1.94)	0.75-11.85
UKB Paired Associate Learning Test	160	7.71 (2.39)	1-10
UKB Tower Test	160	9.79 (3.46)	3-17
UKB Matrix Pattern Test	160	8.86 (2.14)	2-14
General tests			
Self-rated memory	160	0.81 (0.89)	0-3
Mini ACE	159	28.50 (1.88)	20-30
Reference tests			
PPVT	160	219.63 (6.71)	180-227
NART	160	39.94 (6.70)	6-50
NIH Toolbox Picture Vocabulary	160	8.36 (2.27)	0.19-19.40
RMBM Appointments	159	3.10 (1.15)	0-4
WMS-IV Designs I	158	67.09 (13.50)	33-110
WMS-IV Designs II	157	53.69 (11.44)	26-90
WMS-IV Designs Total	157	120.81 (22.71)	67-120
WMS-IV Verbal Paired Associates I	159	34.02 (10.55)	9-54
WMS-IV Verbal Paired Associates II	159	10.04 (2.96)	4-14
WMS-IV Verbal Paired Associates Total	159	72.72 (12.02)	41-94
WAIS-IV Digit Span Forwards	160	11.59 (1.99)	7-16
WAIS-IV Digit Span Backwards	160	10.14 (2.50)	3-16
WAIS-IV Digit Span Sequence	160	8.69 (2.09)	0-14
Symbol Digit Modalities Test	159	50.87 (9.32)	28-81
DLRT Simple Reaction Time	160	344.48 (49.58)	265-508
DLRT Choice Reaction Time	160	584.41 (92.87)	375-901
Trail Making Test part A	160	31.68 (12.35)	16-97
Trail Making Test part B	160	63.31 (26.85)	25-170
D-KEFS Tower Test	160	17.87 (4.08)	2-29
COGNITO Matrices	159	19.77 (4.52)	6-28

UKB, UK Biobank; Mini ACE, Mini Addenbrooke's Cognitive Examination; PPVT, Peabody Picture Vocabulary Test – Fourth Edition; NART, National Adult Reading Test; RMBM, Rivermead Behavioural Memory Test – Extended Version; WMS-IV, Wechsler Memory Scale – Fourth Edition; WAIS-IV, Wechsler Adult Intelligence Scale – Fourth Edition; DLRT, Deary-Liewald Reaction Time Test; D-KEFS, Delis-Kaplan Executive Function System.

[a]For UKB Prospective Memory the value is the number and percentage of participants who were correct on the first attempt.

Supplementary Table 10. Pearson correlations between all cognitive tests with age, sex, education and general health (n = 157-160).

	Age	Sex[a]	Years of education	General health[b]
UK Biobank tests				
UKB Pairs Matching Test	0.339***	-0.100	-0.085	0.025
UKB Reaction Time Test	0.253**	-0.088	-0.040	-0.108
UKB Prospective Memory Test	-0.268**	0.006	0.061	-0.071
UKB Fluid Intelligence Test	-0.229**	-0.017	0.113	-0.015
UKB Numeric Memory Test	-0.207**	-0.005	0.080	0.012
UKB TMT part A	0.575***	0.025	-0.178*	-0.103
UKB TMT part B	0.565***	0.005	-0.213**	-0.025
UKB Symbol Digit Test	-0.598***	0.004	0.107	0.100
UKB Picture Vocabulary Test	0.181*	-0.117	0.291***	0.096
UKB Paired Associate Learning Test	-0.163*	-0.282***	0.052	-0.073
UKB Tower Test	-0.454***	0.186*	-0.043	-0.133
UKB Matrix Pattern Test	-0.474***	0.167*	0.323***	0.104
General tests				
Self-rated memory	0.310***	0.097	0.041	-0.008
Mini ACE	-0.210**	-0.194*	0.146	0.039
Reference tests				
PPVT	0.058	0.030	0.263**	0.069
NART	0.255**	-0.149	0.161*	0.083
NIH Toolbox Picture Vocabulary	0.143	-0.161*	0.285***	0.126
RMBM Appointments	-0.074	-0.074	0.072	-0.099
WMS-IV Designs Total	-0.505***	-0.025	0.124	-0.108
WMS-IV VPA Total	-0.256**	-0.303***	0.140	-0.086
WAIS-IV Digit Span Forwards	-0.120	0.076	-0.016	0.040
WAIS-IV Digit Span Backwards	-0.128	0.035	0.113	0.017
WAIS-IV Digit Span Sequence	-0.167*	0.059	0.070	0.003
Symbol Digit Modalities Test	-0.576***	-0.006	0.150	0.210**
DLRT Simple Reaction Time	0.183*	-0.089	-0.092	-0.178*
DLRT Choice Reaction Time	0.445***	-0.064	-0.193*	-0.231**
TMT part A	0.460***	-0.059	-0.002	-0.077
TMT part B	0.481***	0.028	-0.136	-0.048
D-KEFS Tower Test	-0.277***	0.171*	0.058	-0.036
COGNITO Matrices	-0.334***	0.133	0.241**	0.035

UKB, UK Biobank; TMT, Trail Making Test; Mini ACE, Mini Addenbrooke's Cognitive Examination; PPVT, Peabody Picture Vocabulary Test – Fourth Edition; NART, National Adult Reading Test; RMBM, Rivermead Behavioural Memory Test – Extended Version; WMS-IV, Wechsler Memory Scale – Fourth Edition; VPA, Verbal Paired Associates; WAIS-IV, Wechsler Adult Intelligence Scale – Fourth Edition; DLRT, Deary-Liewald Reaction Time Test; D-KEFS, Delis-Kaplan Executive Function System.

$^{*}p < .05$, $^{**}p < .01$, $^{***}p < .001$

[a]Coded 0 for female, 1 for male.

[b]Coded 1 for poor, 2 for fair, 3 for good, 4 for very good, 5 for excellent.

Supplementary Table 11. Spearman correlations between all cognitive tests with age, sex, education and general health (n = 157-160).

	Age	Sex[a]	Years of education	General health[b]
UK Biobank tests				
UKB Pairs Matching Test	0.296***	-0.145	-0.080	0.010
UKB Reaction Time Test	0.274***	-0.115	-0.030	-0.106
UKB Prospective Memory Test	-0.286***	0.006	0.047	-0.075
UKB Fluid Intelligence Test	-0.267**	0.032	0.105	0.011
UKB Numeric Memory Test	-0.210**	0.007	0.066	0.034
UKB TMT part A	0.622***	0.028	-0.158*	-0.040
UKB TMT part B	0.665***	-0.066	-0.195*	-0.081
UKB Symbol Digit Test	-0.614***	-0.017	0.090	0.077
UKB Picture Vocabulary Test	0.212**	-0.152	0.265**	0.060
UKB Paired Associate Learning Test	-0.171*	-0.302***	0.020	-0.155
UKB Tower Test	-0.465***	0.193*	-0.056	-0.134
UKB Matrix Pattern Test	-0.474***	0.165*	0.262**	0.068
General tests				
Self-rated memory	0.323***	0.076	0.013	-0.013
Mini ACE	-0.208**	-0.190*	0.131	0.012
Reference tests				
PPVT	0.007	0.036	0.219**	0.028
NART	0.247**	-0.151	0.123	0.070
NIH Toolbox Picture Vocabulary	0.139	-0.136	0.255**	0.062
RMBM Appointments	-0.068	-0.072	0.077	-0.089
WMS-IV Designs Total	-0.500***	-0.022	0.118	-0.137
WMS-IV VPA Total	-0.247**	-0.294***	0.140	-0.089
WAIS-IV Digit Span Forwards	-0.128	0.071	-0.055	0.068
WAIS-IV Digit Span Backwards	-0.130	0.049	0.064	0.007
WAIS-IV Digit Span Sequence	-0.178*	0.062	0.014	0.001
Symbol Digit Modalities Test	-0.584***	-0.024	0.124	0.179*
DLRT Simple Reaction Time	0.180*	-0.109	-0.071	-0.180*
DLRT Choice Reaction Time	0.486***	-0.049	-0.213**	-0.219**
TMT part A	0.526***	-0.057	-0.020	0.009
TMT part B	0.570***	-0.059	-0.186*	-0.109
D-KEFS Tower Test	-0.278***	0.187*	0.058	-0.044
COGNITO Matrices	-0.334***	0.142	0.191*	0.006

UKB, UK Biobank; TMT, Trail Making Test; Mini ACE, Mini Addenbrooke's Cognitive Examination; PPVT, Peabody Picture Vocabulary Test – Fourth Edition; NART, National Adult Reading Test; RMBM, Rivermead Behavioural Memory Test – Extended Version; WMS-IV, Wechsler Memory Scale – Fourth Edition; VPA, Verbal Paired Associates; WAIS-IV, Wechsler Adult Intelligence Scale – Fourth Edition; DLRT, Deary-Liewald Reaction Time Test; D-KEFS, Delis-Kaplan Executive Function System.

*$p < .05$, **$p < .01$, ***$p < .001$

[a]Coded 0 for female, 1 for male.

[b]Coded 1 for poor, 2 for fair, 3 for good, 4 for very good, 5 for excellent.

Supplementary Table 12. Spearman rank-order correlations between UK Biobank tests, general tests and reference tests (n = 154-160).

	UKB Pairs matching	UKB RT	UKB Prosp Memory	UKB Fluid IQ	UKB Numeric Memory	UKB TMT part A	UKB TMT part B	UKB Symbol Digit	UKB Picture Vocabulary	UKB PAL	UKB Tower Test	UKB Matrices
General tests												
Self-rated memory	0.141	0.135	-0.143	-0.155	-0.165*	0.310***	0.240**	-0.226**	0.018	-0.039	-0.049	-0.067
M-ACE	-0.228**	-0.064	0.225**	0.314***	0.258**	-0.251**	-0.291***	0.269**	0.359***	0.338***	0.186*	0.187*
Reference tests												
PPVT	-0.169*	-0.150	0.260**	0.352***	0.133	-0.045	-0.102	0.032	**0.721*** **	0.269**	0.120	0.159*
NART	-0.044	0.029	0.082	0.246**	0.097	0.082	0.062	-0.160*	**0.728*** **	0.300***	-0.090	-0.007
NIH TB Picture Vocabulary	-0.107	-0.054	0.176*	0.318***	0.061	0.033	0.053	-0.074	**0.892*** **	0.333***	0.003	0.099
RMBM Appointments	-0.052	-0.093	**0.210** **	0.159*	0.111	-0.100	-0.154	0.133	0.128	0.208**	0.157*	0.093
WMS-IV Designs Total	**-0.319*** **	-0.247**	0.367***	0.318***	0.281***	-0.404***	-0.500***	0.489***	-0.027	0.368***	0.454***	0.457***
WMS-IV VPA Total	-0.258**	-0.034	0.343***	0.175*	0.325***	-0.195*	-0.272**	0.202*	0.320***	**0.467*** **	0.158*	0.245**
WAIS-IV Digit Span Forwards	-0.018	-0.094	0.246**	0.388***	**0.418*** **	-0.186*	-0.259**	0.144	0.134	0.033	0.253**	0.186*
WAIS-IV Digit Span Backwards	-0.057	-0.134	0.264**	0.426***	**0.503*** **	-0.212**	-0.243**	0.190*	0.259**	0.159*	0.206**	0.167*
WAIS-IV Digit Span Sequence	-0.115	-0.072	0.300***	0.426***	**0.388*** **	-0.265**	-0.344***	0.273***	0.084	0.271**	0.398***	0.243**
SDMT	-0.279***	-0.345***	0.254**	0.407***	0.326***	-0.548***	-0.664***	**0.632*** **	-0.069	0.241**	0.500***	0.455***
DLRT Simple RT	0.126	**0.554*** **	-0.120	-0.227**	-0.288***	0.247**	0.228**	-0.232**	0.047	-0.079	-0.158*	-0.174**
DLRT Choice RT	0.209**	**0.514*** **	-0.287***	-0.298***	-0.252**	0.440***	0.589***	-0.494***	0.027	-0.198*	-0.359***	-0.350***
TMT part A	0.251**	0.275***	-0.263**	-0.170*	-0.306***	**0.534*** **	0.557***	-0.578***	0.288***	-0.141	-0.486***	-0.304***
TMT part B	0.303***	0.333***	-0.328***	-0.361***	-0.459***	0.536***	**0.722*** **	-0.585***	0.085	-0.240**	-0.480***	-0.427***
D-KEFS Tower Test	-0.389***	-0.079	0.281***	0.287***	0.309***	-0.280***	-0.415***	0.402***	-0.088	0.190*	**0.395*** **	0.365***
COGNITO Matrices	-0.393***	-0.160*	0.358***	0.312***	0.287***	-0.350***	-0.383***	0.376***	0.157*	0.135	0.347***	**0.509*** **

Correlations shown in bold show the correlations between the UK Biobank tests and the chosen reference test.

UKB, UK Biobank; RT, Reaction Time; Prosp Memory, Prospective Memory; Fluid IQ, Fluid Intelligence; TMT, Trail Making Test; PAL, Paired Associate Learning; M-ACE, Mini Addenbrooke's Cognitive Examination; PPVT, Peabody Picture Vocabulary Test – Fourth Edition; NART, National Adult Reading Test; NIH TB Picture Vocabulary, NIH Toolbox Picture Vocabulary; RMBM, Rivermead Behavioural Memory Test – Extended Version; WMS-IV, Wechsler Memory Scale – Fourth Edition; VPA, Verbal Paired Associates; WAIS-IV, Wechsler Adults Intelligence Test – Fourth Edition; SDMT, Symbol Digit Modalities Test; DLRT, Deary-Liewald Reaction Time Test; D-KEFS, Delis-Kaplan Executive Function System.

*$p < .05$, **$p < .01$, ***$p < .001$

Supplementary Table 13. Age-adjusted Pearson correlations between UK Biobank tests, general tests, and reference tests (n = 154-160).

	UKB Pairs matching	UKB RT	UKB Prosp Memory	UKB Fluid IQ	UKB Numeric Memory	UKB TMT part A	UKB TMT part B	UKB Symbol Digit	UKB Picture Vocabulary	UKB PAL	UKB Tower Test	UKB Matrices
General tests												
Self-rated memory	0.085	0.069	-0.102	-0.107	-0.096	0.165*	-0.074	-0.070	-0.106	-0.060	0.052	0.103
M-ACE	-0.255**	0.015	0.223**	0.317***	0.311***	-0.165*	-0.231**	0.164*	0.426***	0.449***	0.123	0.139
Reference tests												
PPVT	-0.142	-0.170*	0.293***	0.388***	0.198*	-0.096	-0.199*	0.053	**0.747*** **	0.269**	0.136	0.332***
NART	-0.107	-0.056	0.183*	0.374***	0.224**	-0.177*	-0.222**	0.025	**0.738*** **	0.375***	0.073	0.281***
NIH TB Picture Vocabulary	-0.160*	-0.087	0.283***	0.395***	0.181*	-0.147	-0.138	0.037	**0.822*** **	0.358***	0.143	0.287***
RMBM Appointments	-0.050	-0.120	**0.213** **	0.168*	0.107	-0.052	-0.180*	0.069	0.135	0.220**	0.129	0.060
WMS-IV Designs Total	**-0.189* **	-0.152	0.251**	0.230**	0.241**	-0.138	-0.303***	0.266**	0.112	0.298***	0.287***	0.249**
WMS-IV VPA Total	-0.232**	0.037	0.312***	0.159*	0.293***	-0.081	-0.252**	0.074	0.376***	**0.449*** **	0.084	0.182*
WAIS-IV Digit Span Forwards	-0.028	-0.039	0.211**	0.353***	**0.422*** **	-0.178*	-0.190*	0.117	0.172*	0.028	0.241**	0.149
WAIS-IV Digit Span Backwards	-0.080	-0.021	0.242**	0.416***	**0.501*** **	-0.183*	-0.206*	0.127	0.318***	0.179*	0.199*	0.184*
WAIS-IV Digit Span Sequence	-0.071	-0.045	0.265**	0.441***	**0.401*** **	-0.212**	-0.383***	0.253**	0.241**	0.279***	0.348***	0.259**
SDMT	-0.094	-0.267**	0.131	0.301***	0.304***	-0.202*	-0.322***	**0.446*** **	0.121	0.188*	0.309***	0.206**
DLRT Simple RT	0.030	**0.501*** **	-0.148	-0.220**	-0.299***	0.140	0.155	-0.177*	-0.020	-0.135	-0.129	-0.165*
DLRT Choice RT	-0.034	**0.368*** **	-0.249**	-0.216**	-0.219**	0.231**	0.363***	-0.284***	-0.138	-0.147	-0.171*	-0.189*
TMT part A	0.069	0.126	-0.121	-0.059	-0.197*	**0.240** **	0.326***	-0.343***	0.154	-0.053	-0.226**	-0.018
TMT part B	0.138	0.150	-0.210**	-0.227**	-0.445***	0.257**	**0.547*** **	-0.386***	-0.149	-0.308***	-0.298***	-0.201*
D-KEFS Tower Test	-0.336***	-0.054	0.246**	0.227**	0.198*	-0.153	-0.293***	0.316***	0.108	0.211**	**0.322*** **	0.271**
COGNITO Matrices	-0.302***	-0.076	0.352***	0.337***	0.339***	-0.210**	-0.229**	0.284***	0.336***	0.218**	0.261**	**0.502*** **

Correlations shown in bold show the correlations between the UK Biobank tests and the chosen reference test.
UKB, UK Biobank; RT, Reaction Time; Prosp Memory, Prospective Memory; Fluid IQ, Fluid Intelligence; TMT, Trail Making Test; PAL, Paired Associate Learning; M-ACE, Mini Addenbrooke's Cognitive Examination; PPVT, Peabody Picture Vocabulary Test – Fourth Edition; NART, National Adult Reading Test; NIH TB Picture Vocabulary, NIH Toolbox Picture Vocabulary; RMBM, Rivermead Behavioural Memory Test – Extended Version; WMS-IV, Wechsler Memory Scale – Fourth Edition; VPA, Verbal Paired Associates; WAIS-IV, Wechsler Adults Intelligence Test – Fourth Edition; SDMT, Symbol Digit Modalities Test; DLRT, Deary-Liewald Reaction Time Test; D-KEFS, Delis-Kaplan Executive Function System.
*p < .05, **p < .01, ***p < .001

Supplementary Table 14. Age-adjusted Spearman rank-order correlations between UK Biobank tests, general tests and reference tests (n = 154-160).

	UKB Pairs matching	UKB RT	UKB Prosp Memory	UKB Fluid IQ	UKB Numeric Memory	UKB TMT part A	UKB TMT part B	UKB Symbol Digit	UKB Picture Vocabulary	UKB PAL	UKB Tower Test	UKB Matrices
General tests												
Self-rated memory	0.050	0.048	-0.056	-0.077	-0.100	0.147	0.042	-0.037	-0.055	0.018	0.121	0.103
M-ACE	-0.181*	-0.007	0.174*	0.274**	0.228**	-0.159*	-0.238**	0.183*	0.417**	0.314***	0.105	0.104
Reference tests												
PPVT	-0.179*	-0.162*	0.274***	0.368***	0.142	-0.064	-0.157	0.046	**0.736*** **	0.274***	0.140	0.184*
NART	-0.126	-0.046	0.165*	0.336***	0.164*	-0.095	-0.155	-0.011	**0.714*** **	0.358***	0.029	0.129
NIH TB Picture Vocabulary	-0.156*	-0.101	0.227**	0.374***	0.099	-0.068	-0.077	0.014	**0.891*** **	0.365***	0.077	0.189*
RMBM Appointments	-0.033	-0.076	**0.199***	0.148	0.095	-0.075	-0.166*	0.116	0.146	0.199*	0.142	0.070
WMS-IV Designs Total	**-0.197***	-0.144	0.274**	0.228**	0.210**	-0.137	-0.267**	0.269**	0.092	0.322***	0.291***	0.283***
WMS-IV VPA Total	-0.202*	0.038	0.290***	0.116	0.290***	-0.056	-0.157	0.066	0.389***	**0.445*** **	0.053	0.152
WAIS-IV Digit Span Forwards	0.021	-0.063	0.220**	0.371***	**0.405*** **	-0.137	-0.243**	0.083	0.166*	0.011	0.220**	0.143
WAIS-IV Digit Span Backwards	-0.020	-0.107	0.239**	0.410***	**0.493*** **	-0.169*	-0.242**	0.141	0.296***	0.140	0.166*	0.121
WAIS-IV Digit Span Sequence	-0.066	-0.029	0.264**	0.401***	**0.369*** **	-0.200*	-0.324***	0.211**	0.127	0.249**	0.362***	0.183*
SDMT	-0.135	-0.243**	0.113	0.324***	0.263**	-0.294***	-0.460***	**0.429*** **	0.062	0.178*	0.309***	0.245**
DLRT Simple RT	0.078	**0.536*** **	-0.073	-0.188*	-0.262**	0.175*	0.177*	-0.156*	0.009	-0.050	-0.085	-0.102
DLRT Choice RT	0.078	**0.454*** **	-0.176*	-0.201*	-0.177*	0.201*	0.420***	-0.283***	-0.089	-0.133	-0.172*	-0.155
TMT part A	0.118	0.160*	-0.138	-0.038	-0.236**	**0.310*** **	0.332***	-0.380***	0.212**	-0.061	-0.321***	-0.073
TMT part B	0.171*	0.225**	-0.209**	-0.265**	-0.422***	0.282***	**0.570*** **	-0.362***	-0.045	-0.176*	-0.295***	-0.216**
D-KEFS Tower Test	-0.334***	-0.006	0.219**	0.231**	0.269**	-0.143	-0.344***	0.305***	-0.031	0.151	**0.312*** **	0.276***
COGNITO Matrices	-0.329***	-0.081	0.291***	0.249**	0.237**	-0.193*	-0.228**	0.231**	0.252**	0.086	0.229**	**0.423*** **

Correlations shown in bold show the correlations between the UK Biobank tests and the chosen reference test.
UKB, UK Biobank; RT, Reaction Time; Prosp Memory, Prospective Memory; Fluid IQ, Fluid Intelligence; TMT, Trail Making Test; PAL, Paired Associate Learning; M-ACE, Mini Addenbrooke's Cognitive Examination; PPVT, Peabody Picture Vocabulary Test – Fourth Edition; NART, National Adult Reading Test; NIH TB Picture Vocabulary, NIH Toolbox Picture Vocabulary; RMBM, Rivermead Behavioural Memory Test – Extended Version; WMS-IV, Wechsler Memory Scale – Fourth Edition; VPA, Verbal Paired Associates; WAIS-IV, Wechsler Adults Intelligence Test – Fourth Edition; SDMT, Symbol Digit Modalities Test; DLRT, Deary-Liewald Reaction Time Test; D-KEFS, Delis-Kaplan Executive Function System.
*$p < .05$, **$p < .01$, ***$p < .001$

Supplementary Results

Sensitivity analysis

The UKB Picture Vocabulary and UKB Matrices tests shared items with the NIH Toolbox Picture Vocabulary and the COGNITO Matrices tests. To check whether the high correlation found between *g:reference-11* and *g:UKB-11* was because of this overlap in items, another measure of general cognitive ability was created using the same tests as in *g:reference-11*, but excluding scores on the COGNITO Matrices and NIH Toolbox Picture Vocabulary (*g:reference-9*). The results of a PCA using 9 reference tests are reported in Supplementary Fig 5 (scree plot of the eigenvalues) and Supplementary Table 15. The correlations between *g:reference-9* and *g:UKB-11* (r = 0.85, p < .001; age-adjusted r = 0.78, p < .001) and between *g:reference-9* and *g:UKB-5* (r = 0.73, p < .001; age-adjusted r = 0.67, p < .001) were similar in size to that found when using *g:reference-11.*

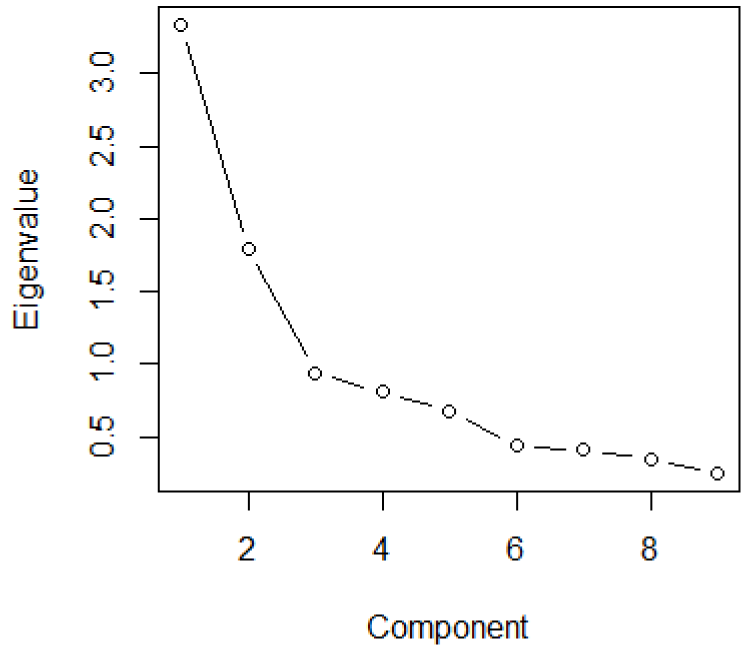

Supplementary Fig 5. Scree plot of eigenvalues from 9 reference cognitive tests (excluding reference tests with overlapping items with the UK Biobank tests).

Supplementary Table 15. Loadings from a principal component analysis of 9 reference cognitive tests (n = 155). Eigenvalues indicated two components. Unrotated and rotated loadings, using oblique (oblimin) rotation, are reported.

	Unrotated PC 1	Unrotated PC 2	Rotated PC 1	Rotated PC 2
Trail Making Test part B	-0.76	0.34	-0.83	0.05
Symbol Digit Modalities Tests	0.75	-0.35	0.83	-0.06
WMS-IV Designs Total	0.74	-0.15	0.73	0.11
WAIS-IV Digit Span Total	0.58	0.25	0.40	0.44
D-KEFS Tower Test	0.53	-0.08	0.51	0.11
DLRT Choice Reaction Time	-0.65	0.33	-0.74	0.08
National Adult Reading Test	0.29	0.86	-0.14	0.92
PPVT	0.45	0.72	0.07	0.84
WMS-IV Verbal Paired Associates Total	0.57	0.29	0.37	0.48
**Proportion of variance**	**0.37**	**0.20**	**0.34**	**0.23**

PC, principal component; WMS-IV, Wechsler Memory Scale – Fourth Edition; WAIS-IV, Wechsler Adult Intelligence Scale – Fourth Edition; D-KEFS, Delis-Kaplan Executive Function System; DLRT, Deary-Liewald Reaction Time; PPVT, Peabody Picture Vocabulary Test – Fourth Edition.

Supplementary Table 16. Spearman rank-order correlations and age-adjusted Spearman rank-order correlations between general cognitive ability, created using 11 reference tests, and the UK Biobank tests (n = 151-160).

	g:reference-11	
	rho	Age-adjusted *rho*
UKB Pairs Matching Test	-0.391***	-0.293***
UKB Reaction Time Test	-0.341***	-0.257**
UKB Prospective Memory Test	0.471***	0.392***
UKB Fluid Intelligence Test	0.555***	0.507***
UKB Numeric Memory Test	0.516***	0.496***
UKB Trail Making Test part A	-0.515***	-0.309***
UKB Trail Making Test part B	-0.672***	-0.534***
UKB Symbol Digit Test	0.558***	0.374***
UKB Picture Vocabulary Test	0.334***	0.509***
UKB Paired Associate Learning Test	0.430***	0.396***
UKB Tower Test	0.521***	0.378***
UKB Matrix Pattern Test	0.544***	0.401***

g:reference-11, measure of general cognitive ability created by entering 11 standardised reference tests into a principal component analysis and saving scores from the first unrotated principal component; UKB, UK Biobank.

**p* < .05, ***p* < .01, ****p* < .001

Supplementary Table 17. Spearman rank-order correlations and age-adjusted Spearman rank-order correlations between two measures of general cognitive ability, created using the UK Biobank cognitive tests, and the general and reference cognitive tests (n = 151-160).

	g:UKB-11		*g:UKB-5*	
	rho	Age-adjusted *rho*	*rho*	Age-adjusted *rho*
Self-rated memory	-0.243**	-0.057	-0.233**	-0.116
M-ACE	0.354***	0.327***	0.332***	0.287***
PPVT	0.246**	0.339***	0.329***	0.370***
National Adult Reading Test	0.015	0.250***	0.128	0.267**
NIH Toolbox Picture Vocabulary	0.111	0.289***	0.209**	0.304***
RMBM Appointments	0.231**	0.256**	0.230**	0.217**
WMS-IV Designs Total	0.655***	0.510***	0.509***	0.401***
WMS-IV VPA Total	0.398***	0.324***	0.353***	0.289***
WAIS-IV Digit Span Forwards	0.373***	0.389***	0.419***	0.408***
WAIS-IV Digit Span Backwards	0.362***	0.391***	0.440***	0.433***
WAIS-IV Digit Span Sequence	0.477***	0.508***	0.471***	0.457***
Symbol Digit Modalities Test	0.692***	0.523***	0.503***	0.374***
DLRT Simple Reaction Time	-0.318***	-0.287***	-0.395***	-0.360***
DLRT Choice Reaction Time	-0.554***	-0.385***	-0.452***	-0.330***
TMT part A	-0.564***	-0.368***	-0.407***	-0.262**
TMT part B	-0.697***	-0.546***	-0.564***	-0.454***
D-KEFS Tower Test	0.484***	0.433***	0.422***	0.359***
COGNITO Matrices	0.521***	0.430***	0.437***	0.361***

g:UKB-11, measure of general cognitive ability created by entering 11 UK Biobank cognitive tests into a principal component analysis and saving scores from the first unrotated principal component; *g:UKB-5*, measure of general cognitive ability created by entering the 5 UK Biobank tests administered at the UK Biobank baseline assessment into a principal component analysis and saving scores form the first unrotated principal component; M-ACE, Mini Addenbrooke's Cognitive Examination; PPVT, Peabody Picture Vocabulary Test – Fourth Edition; RMBM, Rivermead Behavioural Memory Test – Extended Version; WMS-IV, Wechsler Memory Scale – Fourth Edition; VPA, Verbal Paired Associates; WAIS-IV, Wechsler Adult Intelligence Scale, Fourth Edition; DLRT, Deary-Liewald Reaction Time Test; TMT, Trail Making Test; D-KEFS, Delis-Kaplan Executive Function System.

*$p < .05$, **$p < .01$, ***$p < .001$

Supplementary Table 18. Number (%) of participants who answered “no” to whether they thought the UK Biobank cognitive test instructions were clear (n = 145).

	Answered “no”	
UK Biobank test	n	%
UKB cognitive test instructions in general	8	5.5
UKB Pairs Matching Test	5	3.4
UKB Reaction Time Test	3	2.1
UKB Prospective Memory Test	12	8.3
UKB Fluid Intelligence Test	11	7.6
UKB Numeric Memory Test	5	3.4
UKB Trail Making Test	12	8.3
UKB Symbol Digit Test	14	9.7
UKB Picture Vocabulary Test	2	1.4
UKB Paired Associate Learning Test	8	5.5
UKB Tower Test	35	24.1
UKB Matrix Pattern Test	1	0.7

UKB, UK Biobank.

Supplementary references

1. Reitsan RM, Wolfson D. The Halstead-Reitan Neuropsychological Test Battery. Tucson, Arizona: Neuropsychology Press; 1985.

2. Wechsler D. WAIS-IV Administration and Scoring Manual. London: Pearson; 2010.

3. Smith A. Symbol Digit Modalities Test: Manual. Torrance, CA: Western Psychological Services; 1973.

4. Weintraub S, Dikmen SS, Heaton RK, Tulsky DS, Zelazo PD, Bauer PJ, et al. Cognition assessment using the NIH Toolbox. Neurology. 2013;80(11 Suppl 3): S54-64. doi: 10.1212/WNL.0b013e3182872ded.

5. Cooper C. Test the Nation: The IQ Book. London: BBC Books; 2003.

6. Hampshire A, MacDonald A, Owen AM. Hypoconnectivity and hyperfrontality in retired American football players. Sci Rep. 2013;3: 2972. doi: 10.1038/srep02972.

7. Ritchie K, de Roquefeuil G, Ritchie CW, Besset A, Poulin V, Artero S, et al. COGNITO: Computerised Assessment of Information Processing. J Psychol Psychother. 2014;4:2: 1000136. doi: 10.4172/2161-0487.1000136.

8. Roth M, Huppert FA, Tym E, Mountjoy CQ. CAMDEX-R: The Cambridge Examination for Mental Disorders of the Elderly. Cambridge: Cambridge University Press; 1998.

9. Hsieh S, McGrory S, Leslie F, Dawson K, Ahmed S, Butler CR, et al. The Mini-Addenbrooke's Cognitive Examination: a new assessment tool for dementia. Dement Geriatr Cogn Disord. 2015;39(1-2): 1-11. doi: 10.1159/000366040.

10. Hsieh S, Schubert S, Hoon C, Mioshi E, Hodges JR. Validation of the Addenbrooke's Cognitive Examination III in frontotemporal dementia and Alzheimer's disease. Dement Geriatr Cogn Disord. 2013;36(3-4): 242-50. doi: 10.1159/000351671.

11. Folstein MF, Robins LN, Helzer JE. The mini-mental state examination. Arch Gen Psychiatry. 1983;40(7): 812. doi: 10.1001/archpsyc.1983.01790060110016.

12. Dunn LM, Dunn DM. Peabody Picture Vocabulary Test, Fourth Edition: Manual. Bloomington, Indiana: Pearson; 2007.

13. Nelson HE, Willison J. National Adult Reading Test (NART): Nfer-Nelson Windsor; 1991.

14. McGurn B, Starr JM, Topfer JA, Pattie A, Whiteman MC, Lemmon HA, et al. Pronunciation of irregular words is preserved in dementia, validating premorbid IQ estimation. Neurology. 2004;62(7): 1184-6.

15. Dykiert D, Deary IJ. Retrospective validation of WTAR and NART scores as estimators of prior cognitive ability using the Lothian Birth Cohort 1936. Psychol Assess. 2013;25(4): 1361-6. doi: 10.1037/a0033623.

16. Wilson BA, Clare L, Cockburn J, Baddeley A, Tate R, Watson P. The Rivermead Behavioural Memory Test - Extended Version. Bury St. Emunds, England: Thames Valley Test Company; 1999.

17. Wechsler D. WMS-IV Administration and Scoring Manual. London: Pearson; 2010.

18. Deary IJ, Liewald D, Nissan J. A free, easy-to-use, computer-based simple and four-choice reaction time programme: the Deary-Liewald reaction time task. Behav Res Methods. 2011;43(1): 258-68. doi: 10.3758/s13428-010-0024-1.

19. Delis DC, Kaplan E, Kramer JH. Delis-Kaplan Executive Function System (D-KEFS) Examiner's Manual. San Antonio: The Psychological Corporation; 2001.
